# Interfacial Adhesion and Mechanical Properties of PET Fabric/PVC Composites Enhanced by SiO_2_/Tributyl Citrate Hybrid Sizing

**DOI:** 10.3390/nano8110898

**Published:** 2018-11-01

**Authors:** Dandan Pu, Fuyao Liu, Yubing Dong, Qingqing Ni, Yaqin Fu

**Affiliations:** 1 Key Laboratory of Advanced Textile Materials and Manufacturing Technology Ministry of Education, Zhejiang Sci-Tech University, Hangzhou, Zhejiang 310018, China; ddpu0301@163.com (D.P.); liufuyaoer@outlook.com (F.L.); dyb19831120@zstu.edu.cn (Y.D.); niqq@shinshu-u.ac.jp (Q.N.); 2 School of Textiles, Henan University of Engineering, Zhengzhou, Henan 450007, China

**Keywords:** hybrid sizing, surface modification, interfacial adhesion, PET fabric/PVC composites

## Abstract

Poly(ethylene terephthalate) (PET) fabric-reinforced polyvinyl chloride (PVC) composites have a wide range of applications, but the interface bonding of PET fabric/PVC composites has remained a challenge. In this work, a new in-situ SiO_2_/tributyl citrate sizing agent was synthesized according to the principle of “similar compatibility.” The developed sizing agent was used as a PET surface modifier to enhance the interfacial performance of PET fabric/PVC composites. The morphology and structure of the PET filaments, the wettability and tensile properties of the PET fabric, the interfacial adhesion, and the tensile and tearing properties of the PET fabric/PVC composites were investigated. Experimental results showed that many SiO_2_ nanoparticles were scattered on the surface of the modified PET filaments. Moreover, the surface roughness of the modified PET filaments remarkably increased in comparison with that of the untreated PET filaments. The contact angle of the modified PET filaments was also smaller than that of the untreated ones. The peeling strength of the modified PET fabrics/PVC composites was 0.663 N/mm, which increased by 62.50% in comparison with the peeling strength of the untreated ones (0.408 N/mm). This work provides a new approach to the surface modification of PET and improves the properties of PET fabric/PVC composites.

## 1. Introduction

In the past few decades, polyvinyl chloride (PVC)-coated poly(ethylene terephthalate) (PET) fabrics have been applied in the fields of advertising (lightbox cloth), construction (roof cover), and agriculture, among others because of their strength and modulus, good dimensional stability, and lightweight feature [1,2,3]. However, PET fibers have a high degree of crystallinity [4], and the surface lacks polar groups, resulting in poor chemical activity on the PET surface [5]. Hence, forming a solid interface between the PET fabric and the PVC resin matrix is difficult. The peel strength of the interface between PET fabrics and PVC resin matrices is thus inadequate, leading to a partial separation of PET fabrics and PVC resin films in the application of PVC-coated PET fabrics. This condition seriously affects the service life of these materials [6].

Extensive research has been conducted to overcome the poor composite interface problem. Research [7,8] has pointed out that the addition of isocyanate could significantly improve the interface bonding property of composites in the preparation of fabric/PVC composite materials. However, isocyanates are extremely harmful, that is, they pose a health hazard to workers in the production process, and they release harmful substances, such as formaldehyde, during use [9]. Therefore, isocyanates, which improve the bond properties of composites, need to be urgently replaced. Research has showed that the use of adhesives can improve the bonding strength and mechanical properties of composites, but peel strength, in this case, declines [10]. 

Hence, finding an environmentally friendly and economical way to improve the interfacial properties between PET fabrics and PVC resin is necessary. However, PET fibers present a smooth surface and are chemically inert. To modify the surface properties of PET, many researchers have employed treatments using alkali [11,12,13], plasma [14,15,16], biological enzyme [17,18,19], and graft modification [20,21,22], as well as photochemical reactions [23,24], ultraviolet irradiation [25,26], aminolysis surface modification [27], and so on. Unfortunately, most of these approaches have certain limitations. Improper alkali treatment and aminolysis can seriously damage the mechanical properties of fibers, and result in a large amount of waste water. Plasma, ultraviolet, or irradiation cannot be effective in the long term, and will affect the mechanical properties of fibers, and limit their industrial application. The problem of enzyme treatment is that biological enzymes are greatly influenced by the environment and their activity is difficult to control in practice. Grafts usually introduce extra members into the fibers. Coating or sizing, which has attracted considerable attention in the area of fiber surface treatment, is an effective and convenient solution. Recent studies have reported that coating modification technology can change the surface topography and chemical properties of fibers [28,29] and improve their mechanical and chemical bonding, thereby effectively improving the interfacial properties of the composites. Dong et al. reported that the novel water-based hybrid sizing agent synthesized by in-situ generated silica within waterborne epoxy/waterborne epoxy curing agent system can significantly enhance the interfacial shear strength of single glass fiber/epoxy composites [28]. Liu et al. prepared an aqueous epoxy sizing agent by synthesizing a modified epoxy emulsifier to treat carbon fibers (CF) and the interfacial shear strength of sized CF/epoxy composites increased by 70%–76% [29]. Lange et al. found that sizing-treated fibers show increased surface roughness, which enhances their interfacial adhesion to the matrix resin [30]. The improvement of interfacial adhesion could be mainly attributed to the chemical bond and the interface mechanical interlock of fibers and matrix. Pu et al. adopted an SiO_2_/shape memory polyurethane (SMPU) hybrid sizing agent to improve the interface bonding with PET fabric and PVC matrix [31]. However, the SiO_2_/SMPU hybrid sizing agent introduced a new component SMPU into the composite system. Although the SiO_2_ hybrid sizing agent improved the interface bonding of PET fabric/PVC composites, it might adversely affect other properties of composites, and the high cost is not conducive to industrial production. 

In the current work, we took the principle of “similar compatibility” as the design idea based on the infiltration theory of composite materials. Tributyl citrate (TBC), which is the plasticizer of the matrix resin, was selected as the main component of the sizing agent, vinyl triethoxysilane A-151 as the coupling agent, tetraethyl orthosilicate (TEOS) as the precursor, p-toluenesulfonic acid (PTSA) as the catalyst, moisture in the air as water sources, and sol-gel prepared SiO_2_/TBC as the hybrid sizing agent. The sizing agent contained TBC, which is compatible with PVC matrix, and SiO_2_ nanoparticles, which can react with active groups of PET on the PET fiber surface. We expect that the sizing agent can form a well-combined interface between PET and PVC resin and thus provide a new method for improving the interface performance of PET fabric/PVC composites. 

## 2. Experimental Section

### 2.1. Materials

**Figure 1:**
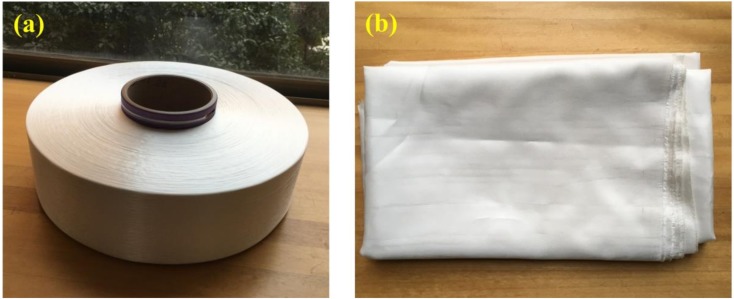
Images of PET filament tows and fabrics used in this work: (**a**) PET filament tows; (**b**) PET filament fabrics.

Kingsway Material Co., Ltd., Haining, China supplied the industrial high-tenacity low-shrinkage PET filament fiber tows (500D/96f) and PET filament fabrics, as shown in Figure 1. The main structure parameters of the PET fabrics are reported in Table 1. The linear density of the warp and weft is the same, and the thread count of the warp and weft is also the same. Thus, the mechanical properties of the warp and weft are almost identical. In this case, the fabric is called a balanced fabric. TEOS, A-151, PTSA, TBC, ethyl alcohol (EtOH), and sodium hydroxide (NaOH) were of analytical grade. Surfactant 1227 and antistatic agent SN were of industrial grade. The main characteristics of PVC resin and epoxidized soybean oil (ESO) are listed in Table 2, as specified by the manufacturer.

### 2.2. Synthesis of SiO_2_/TBC Hybrid Sizing Agent

**Figure 2:**
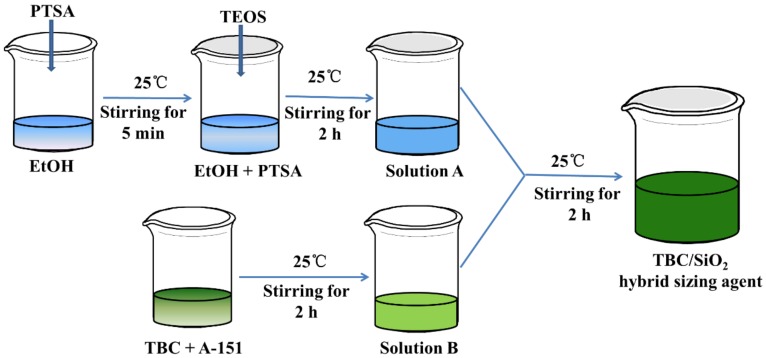
Illustration of the preparation of SiO_2_/TBC hybrid sizing agent.

Solution B was prepared under stirring by mixing TBC and A-151 in a mass ratio of 10:1 at 25 °C for 2 h. The SiO_2_ precursor solution (Solution A) composed of TEOS, PTSA, and EtOH in a mass of 10:0.1:80 was prepared under stirring at 25 °C for 2 h. Solutions A and B were mixed under stirring at 25 °C for 2 h. The mass ratio of A-151/TEOS /TBC was ensured to be 1:5:10. TBC/SiO_2_ hybrid sizing agent was achieved, as shown in Figure 2. Before the application, the TBC/SiO_2_ hybrid sizing agent was diluted with EtOH at a mass ratio of 1:30.

### 2.3. Surface Treatment

#### 2.3.1. Degreasing Treatment

**Figure 3:**
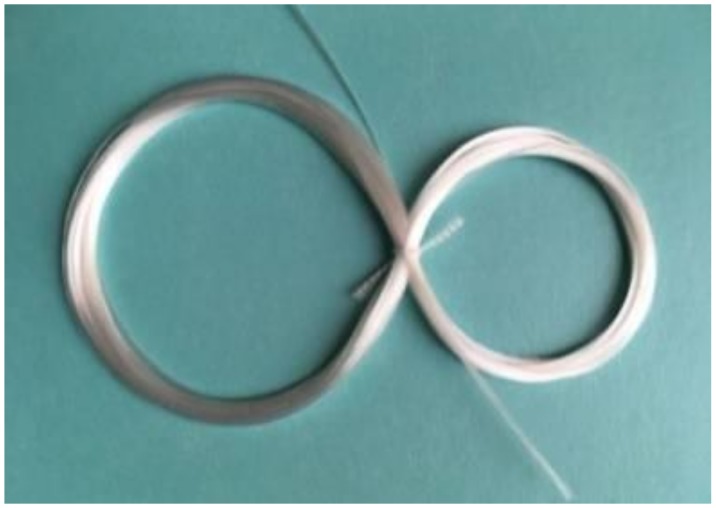
PET industrial filaments used in this study.

The PET industrial filaments were wound into an “8” shape, and the intersection was fixed by a knotted line to avoid entanglement prior to treatments (Figure 3). The PET industrial filaments and PET fabrics were immersed in a mixed solution containing 2 g/L NaOH and 1 g/L SN at 50 °C with a bath ratio of 1:100 for 30 min using a DK-8D electric thermostat tank (Yingmin Instrument Manufacturing Co., Ltd., Changzhou, China). The process was aimed at removing any dirt and residual impurities. Then, the tanks were washed with distilled water repeatedly until neutralized. Finally, the tanks were dried in an oven at 60 °C for 8 h. The samples were labeled as PF.

#### 2.3.2. Alkali Treatment

After the degreasing treatment, the PET industrial filaments or PET fabrics were subjected to alkali treatment. A solution of 10 g/L of NaOH and 1 g/L of Surfactant 1227 in distilled water was prepared, and the filaments or fabrics were soaked in it for 50 min at a temperature of 90 °C while maintaining a bath ratio of 1:100. After the treatment, the filaments or fabrics were rinsed with deionized water several times until their neutral state and then dried in an oven at 60 °C for 8 h. The samples were labeled as PF-1.

#### 2.3.3. Sizing Treatment

Sizing treatment was done via dipping method. The PF-1 filaments or fabrics were stored in a closed container with a diluted TBC/SiO_2_ hybrid sizing agent for 30 min. The samples were then taken out and dried at room temperature for 48 h. The samples were marked as PF-2.

### 2.4. Composite Processing

The hand layup process was used to prepare the composites. First, PVC resin was slowly added to the mixture of TBC and ESO (the weight ratio of the PVC resin plasticizer TBC and stabilizer ESO was 100:130:7), and the resulting mixture was then stirred at 1000 r/min for at least 20 min to achieve a homogeneous paste. A PET fabric was laid flat on Teflon sheets. The prepared paste mixture was poured on the fabric layer and rolled out by a hand roller. The gentle rolling action of the hand roller confirmed the wetting of the PET fabric, and the superfluous paste was squeezed out of the fabric layer by the roller. Then, the composites were cured in an oven at 165 °C for 6 min. The hand layup process was repeatedly conducted after the composites cooled. The thicknesses of all the composites were in the range of 0.85–0.88 mm.

### 2.5. Characterization

#### 2.5.1. Morphology and Structural Characterization of PET Filaments

The surface morphologies of the degreasing (PF), alkali (PF-1), and alkali and sizing-treated (PF-2) PET filaments were observed using a field emission scanning electron microscope (FESEM, Ultra 55, Zeiss, Germany) at an accelerated voltage of 20 kV. The surfaces of the PET filaments were coated with gold through a plasma sputtering apparatus before the FESEM investigation. The PSIA XE-100 atomic force microscope (AFM) was used to observe the surface roughness of the PET filaments. The degreasing-treated PF, alkali-treated PF-1, and alkali and sizing-treated PF-2 were cut into 15 mm segments. Before the test, the samples were washed with alcohol to remove surface contamination and then dried at room temperature. Finally, the samples were scanned with a tapping mode with a scanning area of 2 × 2 μm^2^. Fourier transform infrared spectroscopy (FT-IR) was performed using a Nicolet 5700 FT-IR spectrometer (Thermo Electron Corporation, Madison, WI, USA) to analyze the chemical structures of the PET filaments via the KBr disc technique with a resolution of 4 cm^−1^ in the range of 4000–400 cm^−1^.

#### 2.5.2. Wettability Test

The wettability test for the PET fabric surface was performed with a video contact angle tensiometer (DSA 100 Drop Shape Analysis System, Kruss, Germany). The contact angle values were recorded after 60 ms when the TBC droplet was dropped to the PET fabric. The droplet of the microsyringe was set to 3 μL, and five replications were made. The average value was then calculated.

#### 2.5.3. Tensile Test

The tensile property test for the PET fabrics was conducted in accordance with the GB/T 3923.1-2013 (strip method) of China. The sample length of PET fabric was more than 120 mm, and effective width was 30 mm. Each sample was stretched at a constant rate of 100 mm/min, and the clamping gauge was 100 mm. The test for the PET fabric/PVC composites was carried out according to ASTM D4851: Standard Test Methods for Coated and Laminated Fabrics for Architectural Use under the following conditions: temperature of 21 ± 2 °C and relative humidity of 65% ± 5%. The length of PET fabric/PVC composites specimen was more than 50 mm. The width and thickness were 25 mm and 0.88 mm, respectively. The distance between the holders was 30 mm, and the stretching speed was 100 mm/min. The average value was calculated by five replications.

#### 2.5.4. Peeling Strength Measurement

**Figure 4:**
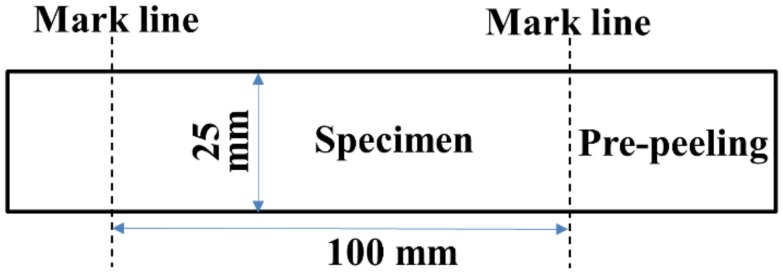
Specimen for peeling strength.

Peeling strength refers to the ratio of the median plateau value of the peeling force to the peeling arm width. According to FZ/T 01010-2012 of China, the test was carried out under the following conditions: temperature of 21 ± 2 °C and relative humidity of 65% ± 5%. The specimen preparation is shown in Figure 4. The peeling strengths of the PET fabric/PVC composites were measured using Instron-3369 at a gauge length of 30 mm and a strain rate of 100 mm/min. The median of the last 80% of the peeling area was counted. Five specimens for each sample were tested. Then, the average values and standard deviations were calculated. 

#### 2.5.5. Tearing Test

**Figure 5:**
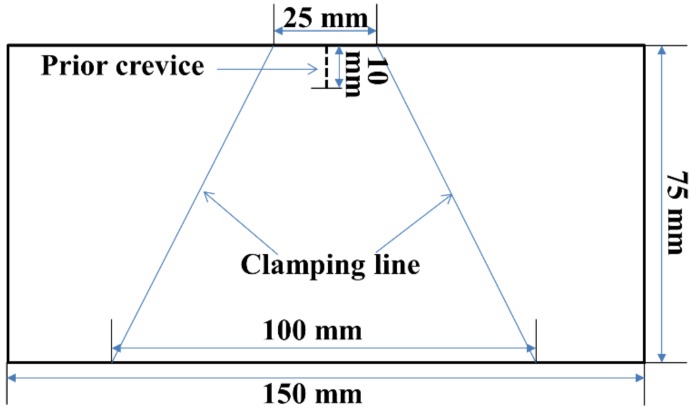
Specimen for tearing strength by trapezoid procedure.

A tearing test was carried out according to ASTM D4851 (Test Method for Tearing Strength by Trapezoid Procedure). Figure 5 shows the specimen preparation for the trapezoid procedure. Tearing tests were carried out under the following conditions: temperature of 21 ± 2 °C and relative humidity of 65% ± 5%. The strain rate was 150 mm/min, and the gap between the two clamps was 25 mm. When the samples were torn off, the average value of the five peaks of the sample tearing curve was taken as the test value. Then, the test value of five replications was calculated as the tearing strength of the sample.

## 3. Results and Discussion

### 3.1. Surface Morphology of PET Filament

**Figure 6:**
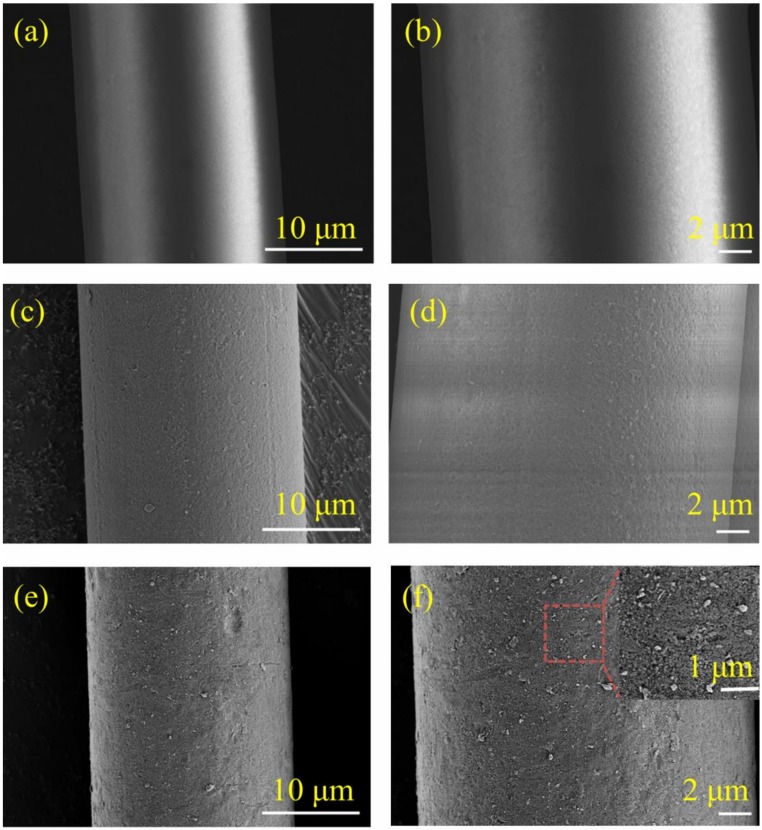
SEM images of PET filaments: (**a**) and (**b**) PF, (**c**) and (**d**) PF-1, (**e**) and (**f**) PF-2.

The surface morphologies of the PET filaments were examined by SEM, as shown in Figure 6. Figure 6a,b show the micrograph of the degreased PF, which was smooth and flat without any impurities deposited on the surface of the filaments. Figure 6c,d show the morphology of PF-1. Dot pits and grooves littered the surface, making it coarse in appearance. The result is due to the etching effect of the alkali treatment on the surface of the PET filaments. The etching effect helps improve the specific surface area and surface activity of PET. Figure 6e,f show numerous nanoparticles scattered on the surface of the sizing-treated PET filaments obtained via the SiO_2_/TBC hybrid sizing agent. The sizing agent was well combined with the PET filaments, and the nanoparticles were semi-embedded in the filaments. The nanoparticles scattered on the filament surface play an important role in increasing the specific surface area.

### 3.2. Surface Roughness of PET Filament

**Figure 7:**
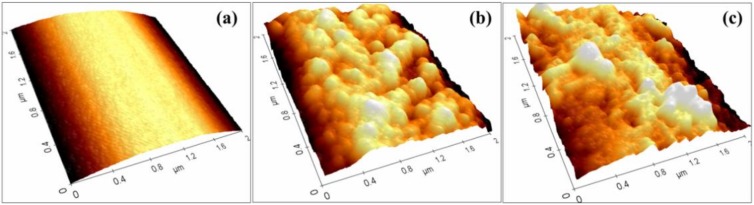
AFM images of PET filaments: (**a**) PF, (**b**) PF-1, (**c**) PF-2.

The surface morphology and surface roughness of PET filaments can be observed precisely with the AFM. Moreover, the surface roughness of the fibers can be calculated. The surface modification changed the surface roughness of the PET filaments. The obtained topography of the PET filaments subjected to different treatments is presented in Figure 7. The degreased PF filament surface was relatively smooth, and the surface of PF-1 filament was uneven. The surface roughness of the PF-1 filament improved to a certain extent. The surface roughness of PF-2 filament improved further due to the surface of the nanoparticles.

The average roughness (*R*_a_) and the root-mean-squared (*RMS*) roughness were used to quantitatively describe the surface roughness. Both values were calculated according to the height of the data points of the AFM images by using the formula (1) and (2).

(1) $$Ra=\frac{1}{n}\sum_{i=1}^{n}\left|h_{i}\right|$$

(2) $$RMS=\sqrt{\frac{1}{n}\sum_{i=1}^{n}h_{i}^{2}}$$

where, *h_i_* is the surface height measured and *n* is the number of surface heights measured.

Changes in the PET surface topography were obvious. The increase in roughness could be seen from the increase in the *R_a_* and *RMS* values, as shown in Table 3. After the alkali treatment, the *Ra* of the PET filaments increased considerably, especially the *RMS*, and the mean value more than doubled from 12.04 nm to 24.24 nm. The PF-1 filaments were sized with a SiO_2_/TBC hybridized sizing agent, and the average value of *RMS* further increased to 39.12 nm possibly because of the nanoparticles deposited on the PET filament surface. This result is in accordance with the surface morphology described above. The fiber surface roughness can improve the mechanical locking of composites.

### 3.3. FT-IR Analysis

**Figure 8:**
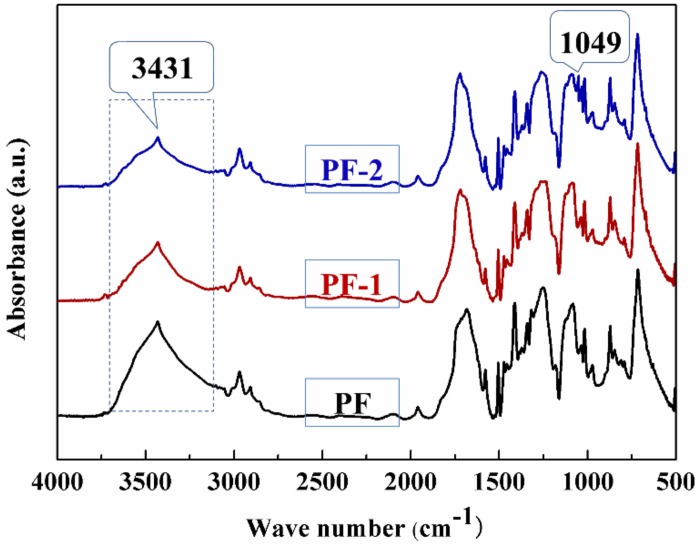
FT-IR spectra of PET filaments.

Figure 8 shows the FT-IR spectra of the PET filaments under different treatments. The peak at 3431 cm^−1^ was due to the stretch vibrations of the hydroxyl in PET. The intensity of the hydroxyl peak of the alkali PET filaments decreased in comparison with that of the degreased PET filaments. Consistent with the literature, this result indicated that the PET filaments hydrolyzed under the alkali treatment [32]. 

For the sizing treatment of the PET filaments, a sharp absorption peak appeared at approximately 1049 cm^−1^, which was ascribed to the characteristic peaks of Si–O–Si created by the formation of sizing through the hydrolysis and condensation of the precursor (TEOS/A-151). The absorption peaks were relatively weak because of the relatively low amount of sizing agents on the PET filaments. A similar case of the characteristic peaks of Si–O was also observed in the surface modification and characterization of aramid fibers with hybrid coating [33].

### 3.4. Wettability of PET Fabrics and TBC

The wettability of fibers is usually characterized by the water contact angle [34]. In the current work, the modification of PET increased its interface bonding with the PVC resin paste containing a certain proportion of plasticizer (TBC). Therefore, the contact angle between the TBC and PET fabric was deemed suitable for characterizing wettability.

**Figure 9:**
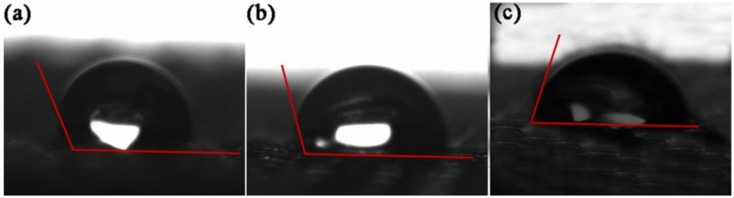
Images of contact angle between TBC and (**a**) PF fabric, (**b**) PF-1 fabric, and (**c**) PF-2 fabric.

Figure 9 show the TBC contact angle for different PET fabrics. The TBC contact angle of the PF fabric (a) was 124.21°, which was higher than that of PF-1 fabric (b) (106.07^o^), indicating that PF-1 fabric was easier to match with TBC because of its large surface area. The TBC contact angle of the PF-2 fabric (c) decreased to 67.73° due to the SiO_2_/TBC hybrid sizing agent, which contained a polar group, thereby increasing the surface tension and freedom of the PF-2 fabrics. Consequently, the PF-2 fabric (c) had excellent compatibility with TBC. According to the wetting theory of composite interfaces, a decrease in contact angle indicates an improvement of the compatibility of the PET fabric with the PVC matrix system. This contributes to the interfacial bonding properties of composite materials.

### 3.5. Tensile Properties of PET Fabrics 

**Figure 10:**
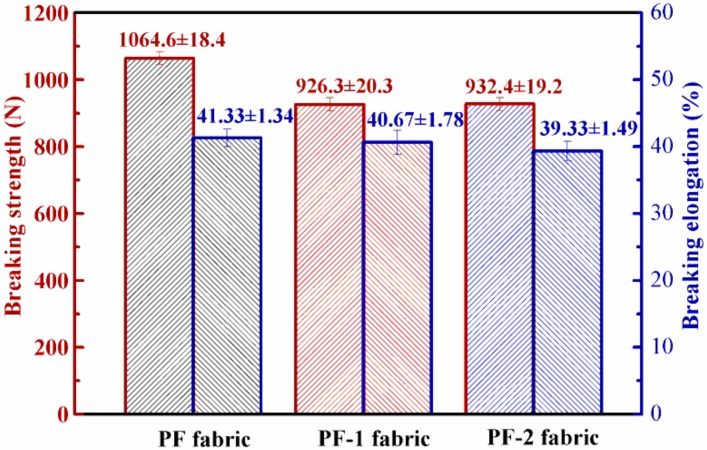
Tensile properties of PET fabrics.

The effects of the treatment on the tensile properties of the PET filament fabrics were determined using a tensile test (Figure 10). The average tensile breaking strength of the PET fabrics decreased from 1064.6 N to 926.3 N because of the alkali treatment. The mean breaking elongation also declined from 41.33% to 40.67%. In addition, the standard deviations of the tensile breaking strength and breaking elongation slightly increased. The data indicated that the alkali treatment damaged the tensile properties of the PET fabrics to some degree. It can be contributed to the fact that PET hydrolyzed under the action of alkali and some chemical bonds on the fiber surface were broken, resulting in a little reduction of tensile breaking strength and breaking elongation. There was no significant difference in tensile breaking strength and breaking elongation between PF-1 fabric and PF-2 fabric. The above statements indicated that the sizing treatment had no adverse effect on the tensile properties of the PET fabrics.

### 3.6. Interfacial Bond Performance of PET Fabric/PVC Composites

**Figure 11:**
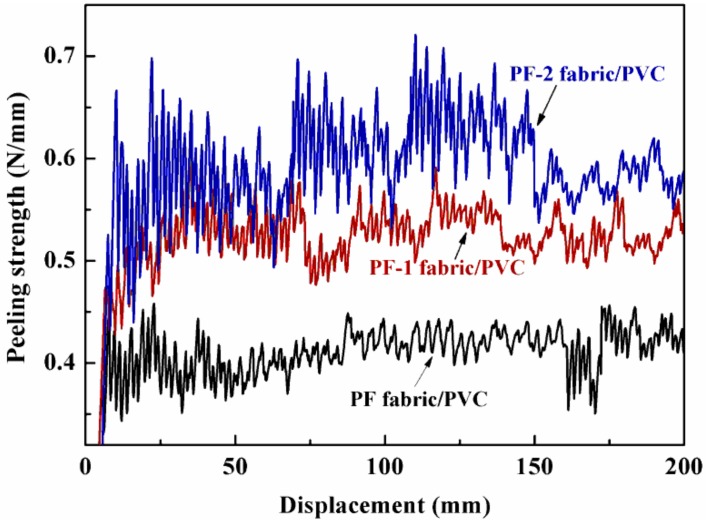
Typical peeling curves of PET fabric/PVC composites.

In the present work, the interface bond strength of the PET fabric/PVC composites was characterized by peeling strength and then studied via the morphology of the cross section. The peeling strength of the PET fabric/PVC composites is shown in Figure 11. The uneven peel strength of the PF-2 fabric/PVC composites was 0.663 N/mm, which was higher than that of the PF-1 fabric/PVC composites (0.537 N/mm). The peeling strength of the PF-2 fabric/PVC composites showed a notable improvement of 62.50% in comparison with the PF fabric/PVC composites.

Figure 11 also shows that the stripping curve of the PF-2 fabric/PVC composites fluctuated more dramatically than the other curves did. It indicated that the peeling strength of the PF-2 fabric/PVC composites was more uneven than the others. This phenomenon can be contributed to the uneven distribution of the cross-linking point in the interfaces of the PF-2 fabric/PVC composites.

On the one hand, TBC as a plasticizer in matrix materials and TBC as a sizing agent of PET fabrics showed the same infiltration or miscibility, which led to a close contact between the PET fabrics and the PVC resin. The interface between the molecules has considerable van der Waals force, which improves the interface binding force. Meanwhile, the interface performance of the composites was effectively enhanced because of the presence of nanoparticles on the interface layer, which increased the friction effect between the interfaces. 

**Figure 12:**
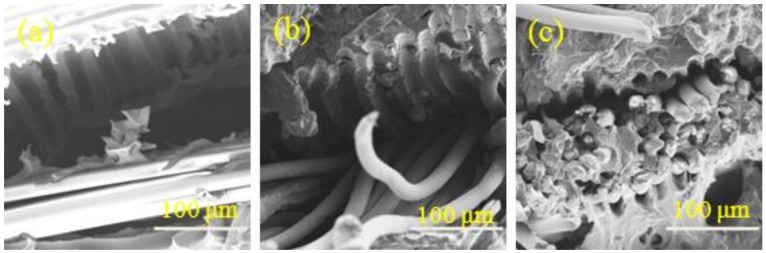
Cross section of (**a**) PF fabric/PVC, (**b**) PF-1 fabric/PVC, and (**c**) PF-2 fabric/PVC composites.

The SEM images in Figure 12 present intuitional and detailed information about the morphology of the cross section of the PET fabric/PVC composites. Figure 12a shows that the fabric was completely pulled out of the matrix and that traces of the fabric were clearly left on the interface of the composites. The fabric and matrix then showed a debonding, which indicates a poor bonding of fabric and matrix. The result could be attributed to the smooth surface of the untreated PET that led to low bonding strength with the matrix. The PF-1 fabric was almost separated from the substrate on the fractured surface of the composites, as shown in Figure 12b. However, the fractured fabric remained on the substrate, thereby revealing that the rough surface of the alkali-treated PET fabrics increased the contact area of the fabrics and the matrix. This condition improved the interface bonding of the composites to a certain extent. Figure 12c shows that the combination of fabric and matrix was satisfactory in the fractured surface of the composites. Sizing treatment increased the surface area of the matrix-impregnated fibers, improved wettability, and increased the active interaction point between the fiber surface and the PVC matrix. This phenomenon enhanced the mechanical interlock and combination force between the fiber and matrix. The hybrid coating similar to a “bridge” firmly connected the matrix and the fiber [28]. Consequently, the interfacial adhesion improved.

### 3.7. Tensile Properties of PET Fabric/PVC Composites 

**Figure 13:**
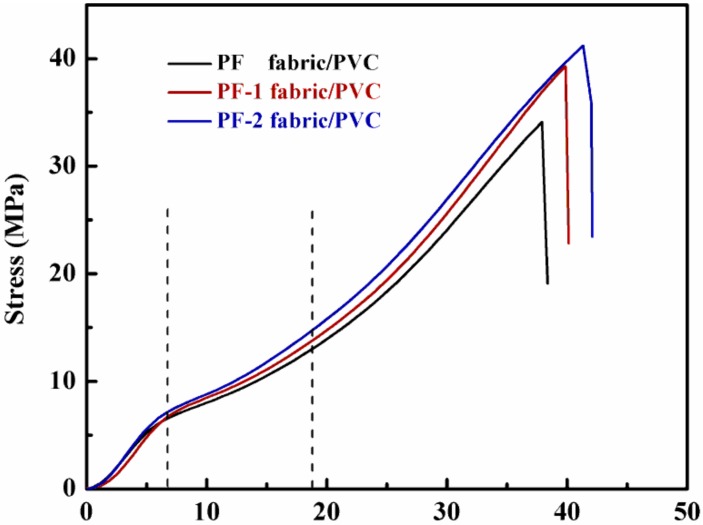
Typical stress–strain curves of the composites.

Figure 13 shows the typical stress–strain curves of the PET fabric/PVC composites. The uniaxial tensile failure process of the PET fabric/PVC composites was divided into three stages. The first stage was the fracture of a few fibers under low stress. The second stage was the spread of the preceding fracture. The last stage was characterized by the eventual destruction of the PET fabric/PVC composites. The three stages were closely related to the performance of the fibers, matrix, and interface. The trend of the stress–strain curves for the three PET fabric/PVC composites maintained a rough consistency. However, in the first stage, the initial modulus of the PF-1 fabric/PVC composites was slightly lower than those of the other composites. 

**Figure 14:**
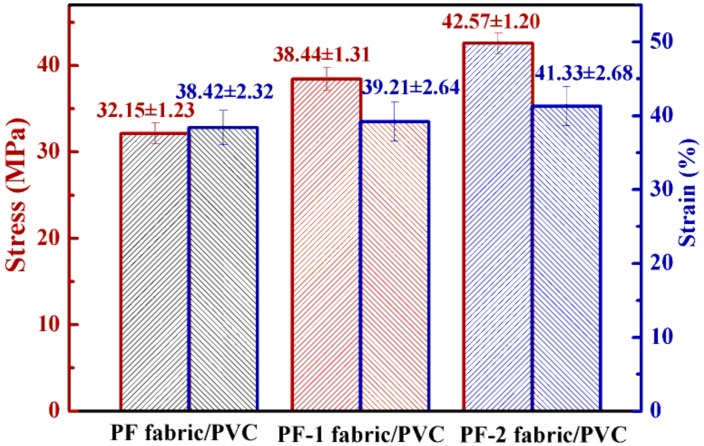
Tensile properties of PET fabric/PVC composites.

When the interface of fabric-matrix composites was enforced by the hybrid sizing agent, the stress was efficiently transferred from the matrix to the hybrid sizing agent and then from the hybrid sizing agent to the fabric [28]. The stress and strain of each studied case are plotted in Figure 14. The PF, PF-1, and PF-2 fabric/PVC composites increased sequentially, thereby indicating the gradual improvement of the interfacial adhesions of the composites. The results were in line with the aforementioned fracture morphology and peeling strength. 

### 3.8. Tearing Properties of PET Fabric/Composites

**Figure 15:**
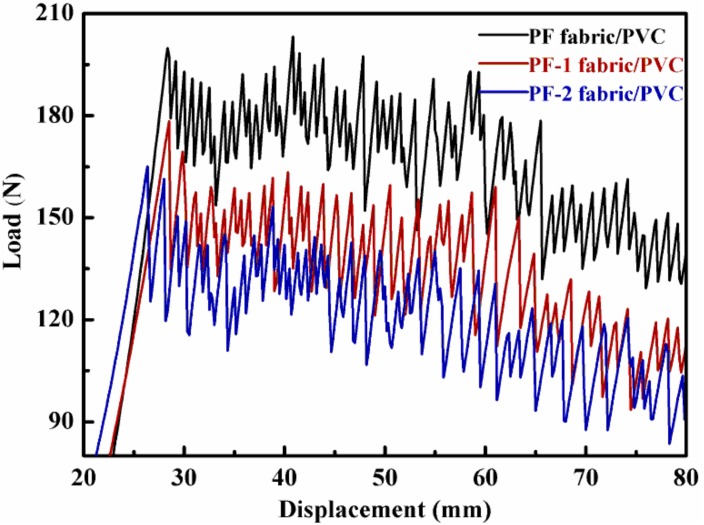
Typical tearing curves of PET fabric/PVC composites.

Tearing performance is an important index for assessing coated fabrics [35]. When a load is applied to a re-cracked sample, a tearing area called the “Del” zone is formed and propagates through the sample [36]. The trapezoidal tear damage is mainly a result of the extension of the “Del” zone in the tearing process [35]. Figure 15 illustrates the variation in the tearing strength of the PET fabric/PVC composites. Following the alkali treatment, the average tearing strength of the PET fabric/PVC composite materials decreased from 173.8 N to 161.2 N. After the next sizing treatment, the average tear strength of the PF-2 fabrics/PVC dropped to 156.8 N, which denoted a decrease of 9.78% in comparison with that of the PF fabric/PVC composites. The decrease in tearing strength was mainly the result of the reduced “Del” zone, which was controlled by the interfacial bond between the PET fabrics and the PVC substrate. Owing to many pits and grooves on the surface of the PF-1 fabrics, there was a good interfacial bond between the PF-1 fabrics and the PVC matrix under the action of mechanical locking. Moreover, the PF-2 fabrics exhibited better bond to the PVC matrix than the PF-1 fabrics due to the presence of nanoparticles and TBC. In the tearing process of the PET fabric/PVC composites, the more interfacial bond of the PET fabric/PVC composites, the more firmly the PET fibers in the fabric were restricted by the PVC matrix, and a smaller “Del” zone was formed. This could explain why the tearing strength of the PF-2 fabric/PVC composites was the lowest, followed by PF-1 fabric/PVC composites and PF fabric/PVC composites. A low tearing strength indicated good interfacial bond strength of the composites. 

In addition, the peak of the tearing curve of PF-2 fluctuated more severely than those of the others. This phenomenon can be ascribed to the uneven distribution of nanoparticles on the interface of the PF-2 fabric/PVC composite materials, as well as to the apparent unbalanced bond between the PF-2 fabrics and PVC substrates. This result is consistent with the surface morphology mentioned above.

## 4. Conclusions

In this study, SiO_2_/TBC hybrid sizing agents were successfully prepared by sol-gel method and then employed as a sizing agent for PET fabrics reinforced with a PVC resin composite system. The results showed that for PET treated by alkali and sizing coating, the surface roughness of the PET filament increased, and the contact angle of the PET fabrics decreased. This condition improved the interfacial bonding performance of PET fabric/PVC composites, with a notable improvement of 62.50% in peeling strength, 32.41% in breaking strength, and 7.57% in elongation at break, whereas the tearing strength was slightly reduced. Therefore, this work gives a new approach to the surface modification of PET and improves the interfacial adhesion and mechanical properties of the PET fabric/PVC composites.

## Figures and Tables

**Table 1:** Structure parameters of PET fabric.

Fabric Weave	Linear Density of Yarn (D/f)		Fabric Count (Threads /10 cm)		Areal Density (g/m^2^)
	Warp	Weft	Warp Count	Weft Count	
Plain weave	500D/96f	500D/96f	120	120	140.03

**Table 2:** Characteristics of PVC resin and ESO.

Characteristics	PVC Resin	ESO
M_w_	168000	1000
M_n_	70000	-
Polydispersity	1450 ± 200	-
Viscosity (Pa·s, 25 °C)	-	0.325
Epoxy value (%)	-	6.2

**Table 3:** Surface roughness of PET filaments.

Sample	R_a_ (nm)	RMS (nm)
PF	10.51 ± 0.38	12.04 ± 0.36
PF-1	19.28 ± 0.65	24.24 ± 0.74
PF-2	32.61 ± 1.39	39.12 ± 1.48
